# Oxidative Damage to Nucleic Acids and Benzo(a)pyrene-7,8-diol-9,10-epoxide-DNA Adducts and Chromosomal Aberration in Children with Psoriasis Repeatedly Exposed to Crude Coal Tar Ointment and UV Radiation

**DOI:** 10.1155/2014/302528

**Published:** 2014-08-12

**Authors:** Lenka Borska, Ctirad Andrys, Jan Krejsek, Vladimir Palicka, Marcela Chmelarova, Kvetoslava Hamakova, Jan Kremlacek, Zdenek Fiala

**Affiliations:** ^1^Institute of Pathological Physiology, Faculty of Medicine in Hradec Kralove, Charles University in Prague, 50038 Hradec Kralove, Czech Republic; ^2^Institute of Clinical Immunology and Allergology, Faculty of Medicine in Hradec Kralove, Charles University in Prague, Simkova 870, 50038 Hradec Kralove, Czech Republic; ^3^Institute of Clinical Biochemistry and Diagnosis, Faculty of Medicine in Hradec Kralove, Charles University in Prague, 50038 Hradec Kralove, Czech Republic; ^4^Clinic of Dermal and Venereal Diseases, University Hospital Hradec Kralove, Novy Hradec Kralove, 500 05 Hradec Kralove, Czech Republic; ^5^Institute of Hygiene and Preventive Medicine, Charles University in Prague, Faculty of Medicine in Hradec Kralove, 50038 Hradec Kralove, Czech Republic

## Abstract

The paper presents a prospective cohort study. Observed group was formed of children with plaque psoriasis (*n=19*) treated by Goeckerman therapy (GT). The study describes adverse (side) effects associated with application of GT (combined exposure of 3% crude coal tar ointment and UV radiation). After GT we found significantly increased markers of oxidative stress (8-hydroxy-2′-deoxyguanosine, 8-hydroxyguanosine, and 8-hydroxyguanine), significantly increased levels of benzo[a]pyrene-7,8-diol-9,10-epoxide (BPDE) DNA adducts (BPDE-DNA), and significantly increased levels of total number of chromosomal aberrations in peripheral lymphocytes. We found significant relationship between (1) time of UV exposure and total number of aberrated cells and (2) daily topical application of 3% crude coal tar ointment (% of body surface) and level of BPDE-DNA adducts. The findings indicated increased hazard of oxidative stress and genotoxic effects related to the treatment. However, it must be noted that the oxidized guanine species and BPDE-DNA adducts also reflect individual variations in metabolic enzyme activity (different extent of bioactivation of benzo[a]pyrene to BPDE) and overall efficiency of DNA/RNA repair system. The study confirmed good effectiveness of the GT (significantly decreased PASI score).

## 1. Introduction

Oxidative stress, characterized by a high degree of reactive oxygen species (ROS), is known to induce damage to cellular components including strand breaks and base modifications in nucleic acids [1]. Oxidative damage to nucleic acids has been found to be associated with a variety of diseases including cancer and aging, but the precise mechanisms are still remaining to be elucidated [2, 3].

Psoriasis is a multifactorial relapsing and remitting inflammatory skin disease. The prevalence of psoriasis ranged from 1.4% to 3.3% in North America and Europe [4]. The disease represents about 4% of all dermatoses which originally manifested at 16 years of age or younger, often as plaque psoriasis [5, 6]. Psoriasis can adversely affect quality of life of children [7].

Goeckerman therapy (GT) represents effective treatment of plaque psoriasis in children [8, 9]. GT is based on daily dermal application of pharmaceutical grade crude coal tar (CCT) ointment with subsequent whole body exposure to UV radiation (UVR). Duration of the treatment is usually 2-3 weeks [10]. Retrospective study demonstrated that GT is effective in children and adolescents with moderate to severe psoriasis [9].

The use of GT has recently decreased for several reasons, including suspected mutagenicity/carcinogenicity of CCT [11–13]. The mutagenicity/carcinogenicity of CCT is probably caused by the presence of polycyclic aromatic hydrocarbons (PAHs). Typical representative of mutagenic/carcinogenic PAHs, benzo[a]pyrene (BaP), is metabolized into a highly reactive metabolite benzo[a]pyrene-7,8-diol-9,10-epoxide (BPDE) that is able to bind to the structure of DNA, RNA, and proteins [14, 15]. The complex of reactive metabolite and DNA structure is known as a DNA adduct. In the case of BPDE it is a BPDE-DNA adduct [16]. An alternative PAHs carcinogenic pathway includes the induction of oxidative stress caused by redox cycling between PAHs dihydrodiols and PAH-o-chinons with subsequent formation of various oxidized forms of nucleobases and nucleosides [17, 18].

Children are more susceptible to the harm effects of environmental exposure and medical treatments than adults as their organism is developing [19, 20]. Recent trends in childhood cancers in the USA and Europe seem to confirm children's increased exposures to genotoxic substances [21].

Elevated levels of biomarkers confirm deleterious effects of carcinogenic PAHs on human health and underline the importance of biomarker analysis as a preventive measure in children. Suitable biomarkers include, among others, PAH-DNA adducts, markers of oxidative stress, and chromosomal aberration [19].

Presented study used biomarkers of oxidative stress and genotoxicity for description of adverse (side) effects associated with application of GT (combined exposure of 3% coal tar ointment and UV radiation) in children. Mentioned effects can damage cells, accelerate aging, induce diseases, and shorten life expectancy.

## 2. Materials and Methods

### 2.1. Study Group

The group was formed of children with chronic stable plaque psoriasis, treated by GT at the Clinic of Dermal and Venereal Diseases, University Hospital, Hradec Kralove (Czech Republic). Over the period of two years, we collected data of 19 children. The group consisted of 12 girls and 7 boys (average age of 12 years, age range 5–17 years). Exposure history of the patients was checked by the questionnaire. Regardless of the young age of respondents, we also assessed their smoking habits. Those patients who had significant prior exposure to PAHs and/or artificial UVR were excluded from the monitored group. The study was approved by the Ethics Committee of the University Hospital in Hradec Kralove, Czech Republic. Informed written consent was obtained from parents of each patient.

### 2.2. Goeckerman Therapy (GT)

Within the therapy, dermatological ointment containing 3% of CCT was administered daily overnight on psoriatic lesions. According to the extent of lesions, 17–40% of the total body surface was covered by CCT ointment. Each morning the residues of CCT ointment were removed from the body (using oil bath) and the patients were whole-body irradiated by UVR.

UVR exposure was dosed according to skin condition and in cases of adverse reactions has been temporarily suspended. For this reason, the total duration of GT (days) may not be equivalent to the time of UVR exposure (days). Irradiation time was individual according to the disease status (1–15 min/day). The density of used radiation was 249.75 *μ*W/cm^2^ of UV-B and 131.8 *μ*W/cm^2^ of UV-A (controlled by Sola-Scope 2000 spectrometer; Solatell, UK). Duration of the treatment was modified according to the treatment effectiveness (average duration of 18 days; range of 14–22 days). Detailed individual characteristic of GT is summarized in Table 1. The effectiveness of the therapy was calculated from basic characteristics of actual disease status (erythema, desquamation, and skin infiltration) and expressed as the PASI score (Psoriasis Area and Severity Index) [22]. Calculation of the efficiency of the treatment was performed according the formula (100 − [PASI after GT/PASI before GT] × 100).

### 2.3. Selected PAHs in Pharmaceutical Grade Crude Coal Tar (CCT)

The pooled sample of CCT (0.1973 g) was sonicated (30 min) with 25 mL of n-hexane and subsequently was diluted fifty times. Instrumental analysis of the extract was performed by gas-chromatography-mass detection: GC Agilent HP 6890/MS HP 5975; column HP-5 ms, Agilent, 30 m/0.25 mm/0.25 *μ*m.

### 2.4. Oxidative Damage of DNA and RNA

Samples of blood were collected from the cubital vein a day before the first treatment and immediately after the last procedure of GT (BD Vacutainer sampling tubes). Blood serum was isolated by centrifugation and stored under −70°C until analysis. Repeated thawing and freezing were avoided. The extent of DNA/RNA oxidative damage was evaluated by EIA Kit (Enzyme Immunoassay, Cayman Chemical Company, Michigan, USA). Oxidative damage of DNA and RNA was presented as the sum of three oxidized guanine species in serum: 8-hydroxy-2′-deoxyguanosine from DNA, 8-hydroxyguanosine from RNA, and 8-hydroxyguanine from either DNA or RNA. The level of DNA/RNA oxidative damage was expressed in picograms of all guanine species per milliliter of serum with detection limit of 33 pg of species/mL of serum.

### 2.5. Benzo[a]pyrene-7,8-diol-9,10-epoxide-DNA Adducts (BPDE-DNA Adducts)

EDTA-treated peripheral blood samples were collected a day before the first treatment and immediately after the last procedure of GT. DNA isolation from lymphocytes was performed and samples were subsequently diluted to a concentration of 2 *μ*g of DNA in 1 mL. The level of BPDE-DNA adducts was determined using the standard method OxiSelect BPDE-DNA Adduct ELISA Kit (Cell Biolabs, Inc., San Diego, USA). The results were expressed as nanograms of BPDE-DNA adducts per microgram of DNA with detection limit of 3.9 ng BPDE-DNA/*μ*g of DNA.

### 2.6. Chromosomal Aberration in Peripheral Lymphocytes

Heparin-treated peripheral blood samples were collected a day before the first treatment and immediately after the last procedure of GT and the level of chromosomal aberration in peripheral lymphocytes was evaluated by standardized method [23]. The method is based on microscopic analysis of lymphocytic chromosomes undergoing mitotic metaphase. In each blood sample, 100 of mitotic sets were evaluated. We counted structurally aberrated cells (SAC), numerically aberrated cells (NAC), and total number of aberrated cells (ABC).

### 2.7. Statistical Analysis

Obtained data were analyzed by using MATLAB rel. 2013b software (MathWorks, Inc., Massachusetts, USA). Because the Lilliefors test of normality had rejected the hypothesis of normal distribution, the data were analyzed by the Wilcoxon signed rank test. The possible associations between monitored biomarkers and important factors of exposure were evaluated by using Spearman Rank Order Correlations.

## 3. Results

For analysis of selected PAHs, we used a pooled sample of CCT (from five different samples of CCT collected in different time of the study). The analyses were done for 16 selected PAHs reported by the US EPA standards (US Environmental Protection Agency). The results are listed in Table 2. The total content of 16 selected PAHs was 9.546 mg/g CCT and used dermatological ointment contains 3% of CCT. This means that 100 g of 3% dermatological ointment contained altogether 28.6 mg of 16 selected PAHs. As resulted from analysis of CCT (Table 2), one PAH is sorted into the group of proven carcinogens, one PAH in the group of probably carcinogens, and six PAHs into the group of possibly carcinogens. Remaining PAHs are registered by IARC; however they are not classifiable as to their carcinogenicity to humans.

**Figure 1:**
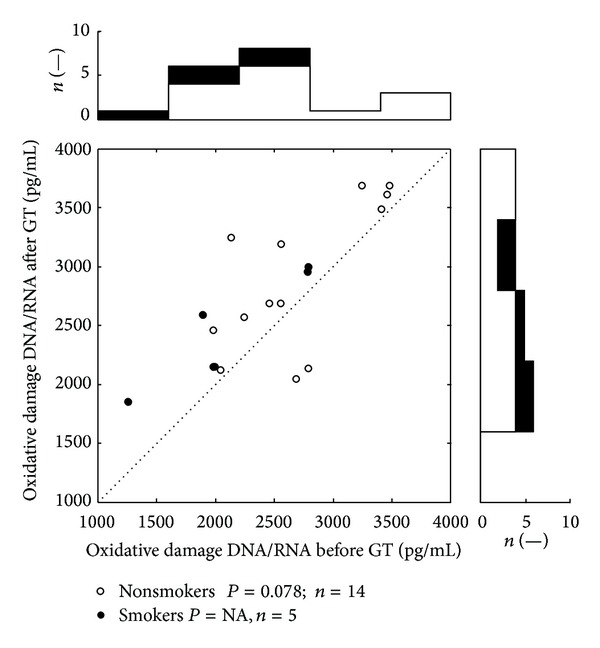
Distribution of values of oxidative damage of DNA and RNA. The oxidative damage of DNA/RNA is presented as the sum of three oxidized guanine species per mL of serum. Scatter plot depicts anti-BPDE-DNA values before and after the GT therapy. All together 19 dots represent 38 measurements; each dot belongs to a one patient. The top histogram shows data distribution before treatment; the right side histogram corresponds to the posttreatment values distribution. The white zones represent nonsmokers data, and the black zones depict smokers' ones. NA: statistically not available.

**Figure 2:**
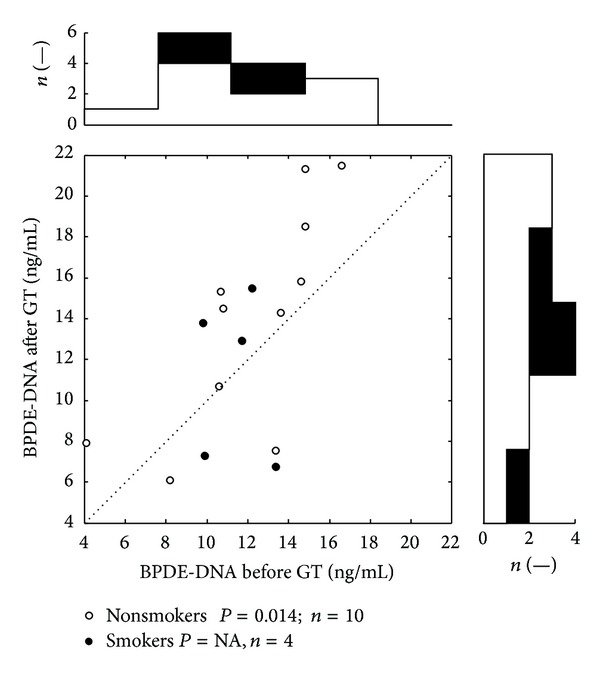
Distribution of values of BPDE-DNA adducts. The levels are expressed as ng of BPDE-DNA adducts per *μ*g of DNA. Scatter plot depicts BPDE-DNA values before and after the GT therapy. All together 14 dots represent 28 measurements; each dot belongs to a one patient. The top histogram shows data distribution before treatment; the right side histogram corresponds to the posttreatment values distribution. The white zones represent nonsmokers data, and the black zones depict smokers' ones. NA: statistically not available.

**Figure 3:**
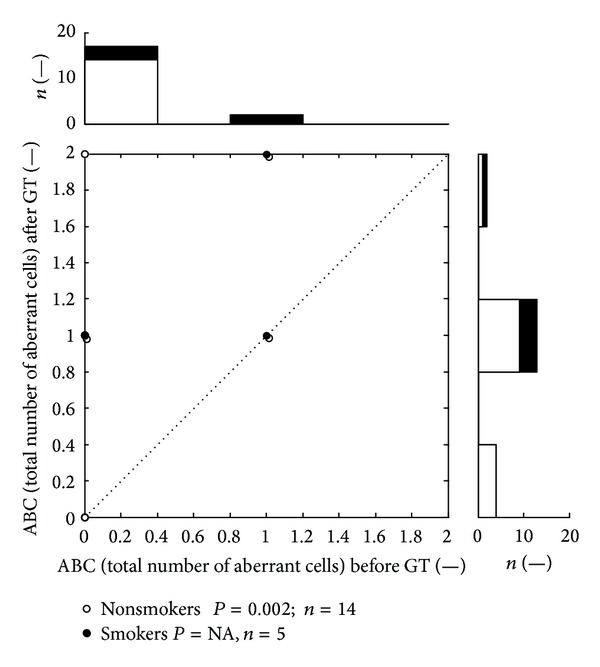
Distribution of values of chromosomal aberration in peripheral lymphocytes (total number of aberrated cells). The levels are expressed as percentage of total number of aberrated cells (ABC). Scatter plot depicts ABC values before and after the GT therapy. All together 5 dots represent 19 subjects and 38 measurements; number of patients belonging to one dot is shown in histograms. The top histogram shows data distribution before treatment; the right side histogram corresponds to the posttreatment values distribution. The white zones represent nonsmokers data, and the black zones depict smokers' ones. NA: statistically not available.

Table 3 summarizes the results of biomonitoring of oxidative stress and genotoxic effects associated with the application of GT in observed group. After GT (3% of CCT) we found significantly increased serum levels of oxidative damage of DNA and RNA (*P* < 0.05), BPDE-DNA adducts (*P* < 0.01), and total number of aberrated cells (*P* < 0.001). Increased levels of biomarkers are clearly visible in the attached scatter plots (Figures 1, 2, and 3) which depicts distribution of values of oxidative damage of DNA and RNA, BPDE-DNA adducts, and chromosomal aberration in peripheral lymphocytes (total number of aberrated cells) before and after the GT. Each dot belongs to at least one patient (number of patients belonging to one dot is depicted in histograms). Values above the dotted diagonal line reflect the increase of the oxidative and genotoxic damage after the therapy. Probability of difference between the pre- and the posttreatment medians was evaluated by Wilcoxon signed rank test. The top histogram shows distribution of followed parameters before treatment; the right side histogram corresponds to the posttreatment values distribution. On these histograms the development of changes in the distribution of values can be well observed.

**Figure 4:**
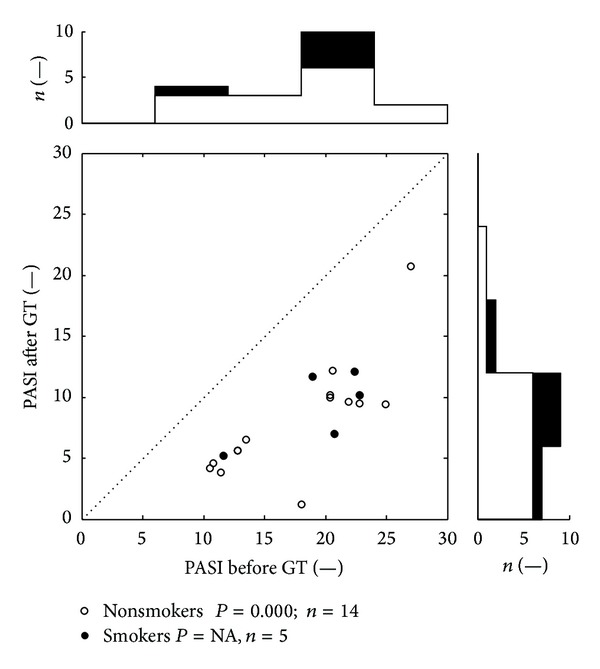
Distribution of PASI score. The levels are dimensionless. Scatter plot depicts PASI score before and after the GT therapy. All together 18 dots represent 38 measurements; each dot belongs at least to a one patient. The top histogram shows data distribution before treatment; the right side histogram corresponds to the posttreatment values distribution. The white zones represent nonsmokers data, and the black zones depict smokers' ones. NA: statistically not available.

The effectiveness of GT was high and the values of PASI score significantly decreased after GT (*P* < 0.001). Before the therapy we found 20.4/12.8–22.3 (median/lower-upper quartile of PASI) and after the therapy 9.4/5.3–10.2 (median/lower-upper quartile of PASI).  Figure 4 depicts the PASI score with a similar layout as Figures 1–3. The values representing positive treatment effect are below the diagonal dotted line. The Wilcoxon's test for smokers for such small group did not have sufficient power to detect differences between pre- and posttreatment values and therefore *P* is reported as not available (NA).

Table 4 presents the analysis of potential associations between monitored biomarkers (values after GT) and important characteristics of exposure. We found significant relationship between (1) time of UV exposure and total number of aberrated cells (*P* < 0.05), (2) daily topical application of CCT ointment (% of body surface) and BPDE-DNA adducts (*P* < 0.05), and (3) daily topical application of CCT ointment (% of body surface) and PASI score (*P* < 0.01).

Cigarette smoking was reported by 5 patients. Number of smoked cigarettes was overall low and irregular and ranged from one to four cigarettes per “ordinary” day (during hospitalization they almost did not smoke at all). Group of respondents who admitted smoking was not statistically evaluable because of low number of the patients (4-5). For the reasons above, the statistical evaluations in Tables 3 and 4 were performed for the whole group of respondents and the values of potential smokers were marked only in Figures 1–4.

## 4. Discussion

The carcinogenicity of individual PAHs and PAHs containing mixtures has been studied in experimental animals. Virtually no data exist on the carcinogenicity of individual PAHs in humans, and only a limited amount of data on the carcinogenicity of PAHs containing mixtures is available for humans. There is evidence that a number of individual PAHs are carcinogenic in experimental animals, while others have been found to be noncarcinogenic. Because of the varying carcinogenic potency of individual PAHs, it is not possible to provide a single weight of evidence carcinogenicity assessment for PAHs as a class. As it was shown in Table 2, the carcinogenicity of each PAHs should be assessed separately [24].

While it is not possible to provide a carcinogenicity assessment for PAHs as a class, it may be possible to evaluate the carcinogenicity of PAHs containing complex mixtures. There is good evidence that some PAHs containing mixtures are carcinogenic to humans (e.g., bitumen, carbon black, coal tars, creosote, and soot). Coal tars are classified according to IARC (International Agency for Research on Cancer) in group 1: the agent is carcinogenic to humans [24].

As can be seen from the results the amount of 100 g of 3% CCT ointment contains 0.024 mg of proved carcinogens and 9.942 mg of possible carcinogens. The average amount of 3% CCT ointment applied to treated skin was approximately 4 mg/cm^2^. Extent of psoriatic lesions ranged approximately from 10 to 80% of the body surface and, thus, corresponding total dose of applied CCT ointment ranged from units to tens of grams. The total amount of carcinogenic PAHs applied to the skin of given patient (and related hazard) can be assumed on the base of these data. As an example we can describe the hazards of some patient with a body surface area of about 14.000 cm^2^ and with the 50% of skin affected by psoriatic lesions (7.000 cm^2^). Then the theoretical total daily dose of 16 selected PAHs will be 8.0 mg and of carcinogenic PAHs 2.7 mg (PAHs from the group of 16). However, although the group of 16 selected PAHs is toxicologically significant and representative it should be noted that the group of selected 16 PAHs represents only a small fraction of the total content of PAHs in the CCT.

It must be noted that the mixture of UV-A and UV-B radiation (part of GT) is also ranked by IARC into Group 1 with definition: ultraviolet radiation, wavelengths 100–400 nm, encompassing UVA, UVB, and UVC. It is evident that both components of the therapy (CCT and UVR) present a relatively high carcinogenic potential. Moreover, in situation of combined exposure to PAHs and UV radiation even their synergistic effects on generation of ROS, lipid peroxidation, and DNA damage can be expected [25, 26].

PAHs may exert their mutagenic, genotoxic, and carcinogenic properties by two major mechanisms. One pathway includes induction of oxidative stress [17, 18, 27]; second pathway includes formation of specific PAHs-DNA adducts [16, 28, 29].

Oxidative stress represents a deregulation of the homeostasis between the reactive oxygen species and the mechanisms of detoxification and repair [27]. The currently available epidemiological and laboratory data indicate that oxidative stress plays a central role in carcinogenic potential effect of PAHs [30]. PAHs can be metabolized by the cytochrome P450 enzymes to active semiquinones, which are known free radicals intermediates and can go through redox cycling (between PAHs dihydrodiols and PAH-o-chinons) and generate reactive oxygen species (ROS) [28]. Subsequently, the ROS can then cause oxidative modification of DNA and lipids in the body [30]. Oxidative damage to nucleic acids has been found to be associated with a variety of diseases, including cancer and aging. Guanine is the base that is the most prone to oxidation. The 8-hydroxy-2′-deoxyguanosine (from DNA) is the form of oxidized guanine that is the most commonly studied critical biomarker of oxidative DNA damage in adults and children exposed to PAHs [31–33].

In recent years, it has become increasingly clear that both DNA and RNA are damaged by oxidation in disease states and that the repair processes that are initiated to correct this damage release multiple oxidized guanine residues, including the ribose free base (8-oxo-guanine or 8-hydroxyguanine), the nucleoside from RNA (8-oxo-guanosine or 8-hydroxyguanosine), and the deoxynucleoside from DNA (8-oxo-deoxyguanosine or 8-hydroxy-2′-deoxyguanosine) [2, 18]. These residues are released into the body fluid, including cerebrospinal fluid, plasma/serum, and urine under the condition of oxidative stress [1, 2].

While 8-hydroxy-2′-deoxyguanosine is the form most researches are familiar with, other published studies have reported that the RNA residue 8-hydroxyguanosine is a better biomarker of age-related oxidant damage [17] and that the base (8-hydroxyguanine) is a better biomarker in some cancer patients [3, 27, 34]. From the reasons described above it is recommended to analyze more than one oxidation (damage) product, since if just one damage product is measured, there is a risk that the specific damage (product) may not be a good marker of the oxidative stress, and thus not give a true picture of the amount of oxidative stress the subject in question is exposed to [35].

It was shown in Table 3 that the level of oxidative damage of DNA and RNA was significantly increased after the therapy (*P* < 0.05). This exposure scenario indicates (in given group of children) increased hazard of oxidative stress related to the treatment. However, it must be noted that although higher level of oxidized guanine residues indicates elevated level of oxidative stress, it can also reflect a high level of efficiency of the processes that work to repair this damage (oxidative stress can be high and the repair processes eliminate its effects). Therefore the interpretation of this bioindicator for the purpose of health risk assessment requires a certain degree of caution.

In the presented study the oxidative damage of DNA and RNA was presented as the sum of three oxidized guanine species in serum: 8-hydroxy-2′-deoxyguanosine from DNA, 8-hydroxyguanosine from RNA, and 8-hydroxyguanine from either DNA or RNA (pg/mL of serum). To the best of our knowledge the state of oxidative stress (expressed as different serum oxidized guanine species) in children dermally exposed to CCT and UVR has not been yet reported. Previously published studies were focused mostly on relationship between inhalation exposure to PAHs and different guanine species in urine (not in serum). For example the study of Rossnerova et al. presented inhalation exposure to benzo[a]pyrene and related urinary 8-oxo-7,8-dihydro-2′-deoxyguanosine in children living in highly polluted area. The level of urinary 8-oxo-7,8-dihydro-2′-deoxyguanosine of these children ranged from 5.96 to 6.73 nmol/mmol creatinine [32]. Other authors described impact of inhalation exposure of children to PAHs (traffic pollution; polluted versus nonpolluted area) on urinary 8-hydroxy-2′-deoxyguanosine [31]. They did not find significant difference for urinary 8-hydroxy-2′-deoxyguanosine concentration between the groups from polluted and nonpolluted areas (20.87 versus 16.78 *μ*mol/mol creatinine). It can be seen that urinary 8-hydroxy-2′-deoxyguanosine concentration was only slightly higher in the group from polluted area. The authors assume that the potential of coexposure of the children to other pollutants affects 8-hydroxy-2′-deoxyguanosine concentration besides the PAHs. However, as stated previously, it cannot be excluded that the used oxidation product (8-hydroxy-2′-deoxyguanosine) was not the most suitable marker of the oxidative stress and thus did not give a real picture of the amount of oxidative stress [35]. On the other hand a clear link between heavy occupational exposure to PAHs (coke oven workers) and the levels of urinary 8-hydroxydeoxyguanosine was found in the work of Kuang et al. [30]. The levels of urinary 8-hydroxydeoxyguanosine ranged from 81.7 (control) to 115.7 (heavily exposed group) nmol/mmol creatinine.

Benzo(a)pyrene is notable for being the first chemical carcinogen to be discovered. BaP is a five-ring PAH known to be procarcinogen. Its mechanism of carcinogenesis is dependent on a three-step enzymatic metabolism to final mutagen. The steps include generation of benzo(a)pyrene-7,8-epoxide, benzo(a)pyrene-7,8-diol, and finally benzo(a)pyrene-7,8-diol-9,10-epoxide (BPDE). Very reactive BPDE binds covalently to proteins, lipids, and DNA (guanine residues) to produce BPDE-DNA adducts. If left unrepaired, DNA adducts may lead to permanent mutations resulting in cell transformation and ultimately to tumor development [15]. Biological monitoring includes indicators of exposure, biological effect, and susceptibility. BPDE-DNA adducts can be indicative of both exposure and genotoxic effect of BaP. The level of BPDE-DNA adducts is determined by the character of BaP metabolism (metabolic enzyme activities) and by the degree of reparation mechanisms.

It was shown in Table 3 that the level of BPDE-DNA adducts was significantly increased after the therapy (5.65 versus 7.2 ng BPDE-DNA adducts/*μ*g DNA; *P* < 0.01) and it indicates increased hazard of genotoxic effects related to the treatment. However, similar to the scenario with oxidative stress, it must be noted that the final level of BPDE-DNA adducts reflects combined effect of intensity of exposure to BaP, metabolic activity (production of BPDE-DNA adducts), and efficiency of DNA/RNA repair mechanisms. The worst scenario should include high level of exposure, high production of BPDE-DNA adducts (high metabolic activity of responsible CYP enzymes), and limited activity of repair system. This indicates (analogously to the biomarkers of oxidative stress) that the interpretation of the level of BPDE-DNA adducts for purposes of health risk assessment requires a certain degree of caution.

The number of epidemiological studies related to BPDE-DNA adducts in children is limited [15, 29, 36]. In addition, these studies are different in used methods, units, and analyzed biological materials. Thus, the comparison of results is a little bit complicated. For example, Pauk et al. compared the levels of total DNA adducts in lymphocytes between different groups of respondents [15]. These groups included healthy persons, patients with chronic obstructive pulmonary disease (noncancerous lung disease which occurs mainly due to smoking and other long-term respiratory exposure to chemical substances), and patients with lung cancer. The level of total DNA adducts was determined using P^32^-postlabeling method and ranged between 0.710 and 1.329 A.U. (arbitrary units). The patients with diseases largely associated with smoking (PAHs) showed increased levels of total DNA adducts in comparison to nonsmoking patients. The influence of tobacco smoke (PAHs) on the level of total DNA adducts and B(a)P-like adducts in newborns and mothers was studied by Topinka et al. [36]. PAH-DNA adducts and B(a)P-like adducts were determined using P^32^-postlabeling method. In subjects unexposed to tobacco smoke, the level of total DNA adducts in lymphocytes ranged from 1.06 (newborns) to 1.13 (mothers) and the level of B(a)P-like adducts ranged from 0.16 (newborns) to 0.19 (mothers) DNA adducts/108 nucleotides. In subjects exposed to tobacco smoke the level of total DNA adducts in lymphocytes ranged from 0.92 (newborns) to 1.18 (mothers) and the level of B(a)P-like adducts ranged from 0.15 (newborns) to 0.22 (mothers) DNA adducts/108 nucleotides.

Genotoxic exposures occurring during childhood may continue for several years, become chronic, and eventually play a relevant role in the etiology of childhood as well as adulthood cancers. Chromosomal aberrations in human peripheral lymphocytes represent well-established biomarker of genotoxicity, probably the only one which has been internationally standardized and validated for children [19, 21, 37].

In our study we found significantly increased levels of ABC (*P* < 0.01) and SAC (*P* < 0.05) after GT and these findings support assumption of elevated genotoxic hazard (Table 3). Before GT we found only two aberrations (ABC) in the total number of 1900 cells (0.10%). One aberration was classified as a structural abnormality (SAC) and one cell as a numerical abnormality (NAC). After GT we found seventeen aberrations (ABC) in the total number of 1900 cells (0.80%). Eleven aberrations were classified as structural abnormalities (SAC) and six as numerical abnormalities (NAC). Declared reference value for the total number of aberrated cells (ABC) of healthy Czech children ranges from 0 to 1.85%. This is a range of values for a group of children (*n* = 20) [23, 38]. From the above it is evident that treatment increased the level of aberrated cells in lymphocytes (genotoxic hazard). Under this condition we can consider that a similar situation of elevated genotoxic hazard was obvious also in other tissues/organs that are well perfused with blood. In conclusion, it must be emphasized that the level of abnormalities which was found after the therapy (0.80%) is still below the upper level of the reference range for healthy Czech children (1.85%; *P* < 0.01). This means that although there was an increase of genotoxic risk, this risk is within the acceptable values.

The results of the present study agree well with the results of our previous work [8]. In that work, we studied a group of children treated with GT using 5% coal tar ointment. After the treatment, there were found significantly increased levels of aberrations (ABC; median 2.0%; lower-upper quartile 2.0-3.0%). As it can be seen, the median value of ABC in the case of foregoing study (5% of CCT) was greater than median value presented in this paper (3% CCT) and exceeded the upper level of the reference range for healthy Czech children (1.85%). The results from both studies suggest possible dose dependency.

Nowadays, it is generally accepted that the high frequency of cytogenetic biomarkers, chromosomal aberrations, can predict an increased risk of genotoxic effect in children after exposure to PAHs. Exposure situations can be described either by simply increasing the levels from initial state (if known) or by comparison with the reference value(s). It is known that cytogenetic biomarkers may vary with gender and age in children (adults). The age extent of children in our group ranged from 5 to 17 years and the group includes boys and girls. It may therefore be the question whether this age range and gender differences can lead to some shifts of reference values of chromosomal aberrations. Merlo et al. analyzed data from 16 published epidemiologic studies performed on pediatric population and from a large sample of Czech children aged 7–16 years. For the whole referent population (age range 0–19 years) the mean frequency of chromosomal aberration was 1.24 (95% CI = 1.05–1.47). Similar baseline levels were found for chromosome breaks frequency in boys and girls: 1.22% and 1.21%, respectively. In conclusion, based on the reviewed studies, baseline levels for chromosomal aberrations were similar in boys and girls and failed to show any increase with age [37].

While it appears that most of genotoxic effects of GT can be attributed to PAHs, it is likely that UVR, as integral part of GT, increases the risk for mutagenicity and carcinogenicity. UVR is known to increase the toxicity of PAHs through photoactivation and photomodification [39]. Skin damage caused by exposure to BaP is further increased by exposure to both UV-A and UV-B radiation. The effects related to UV-A radiation seem to be more significant [40]. It has been shown that BaP, in combination with UV-A radiation, synergistically induced oxidative DNA damage. These facts indicate that UV-A radiation is able to transform BaP into more harmful compounds [39]. Another research demonstrates that BaP in combination with UV-A radiation can substantially increase oxidative damage of DNA via a ROS mechanism (primarily represented as elevated levels of 8-hydroxy-2′-deoxyguanosine) [16, 25, 41]. In the presented work (Table 4) we found significant relationship between time of UV exposure and total number of aberrated cells (*P* < 0.05).

The effectiveness of the therapy was expressed as the PASI score. In the presented study, the PASI score was significantly decreased after GT (*P* < 0.001) and confirmed generally assumed high effectiveness of the GT for children [8, 9]. We found significant relationship between the PASI score and daily topical application of CCT ointment (% of body surface).

## 5. Conclusion

We found significantly increased markers of oxidative stress (8-hydroxy-2′-deoxyguanosine, 8-hydroxyguanosine, and 8-hydroxyguanine), significantly increased levels of BPDE-DNA adducts, and significantly increased levels of total number of chromosomal aberration in peripheral lymphocytes of children with psoriasis treated by GT. These findings can indicate increased hazard of oxidative stress and genotoxic effects related to the treatment. However, it must be acknowledged that the oxidized guanine species and BPDE-DNA adducts also reflect the individual variations in metabolic enzyme activity (different extent of bioactivation of BaP to BPDE) as well as the overall efficiency of DNA/RNA repair system. The study confirmed good clinical effect of the GT (significantly decreased PASI score).

## Figures and Tables

**Table 1:** Detailed characteristic of the treatment.

Number of patients	Total duration of GT (days)	Time of UVR exposure (days)	Daily topical application of CCT ointment (% of body surface)
1	22	18	32
2	17	10	19
3	18	13	37
4	22	22	38
5	22	19	31
6	18	15	20
7	14	9	40
8	14	10	28
9	17	17	31
10	14	13	40
11	19	15	28
12	21	21	37
13	22	22	37
14	14	9	30
15	14	10	17
16	17	17	38
17	22	23	28
18	17	17	36
19	18	18	28

**Table 2:** The content of selected PAHs in pharmaceutical grade crude coal tar (CCT).

PAHs	IARC^a^	mg/g	PAHs	IARC^a^	mg/g
Benzo(a)pyrene	1	0.008	Acenaphthene	3	0.104
Dibenz(a,h)anthracene	2A	0	Anthracene	3	2.494
Benz(a)anthracene	2B	0	Benzo(g,h,i)perylene	3	0
Benzo(b)fluoranthene	2B	0	Fluoranthene	3	0.413
Benzo(k)fluoranthene	2B	0	Fluorene	3	0.299
Chrysene	2B	0.028	Phenanthrene	3	2.520
Indeno(1,2,3-c,d)pyrene	2B	0	Pyrene	3	0.241
Naphthalene	2B	3.286	Acenaphthylene	—	0.153

^a^Classification according to WHO (IARC Monographs on the Evaluation of Carcinogenic Risk to Humans, Volumes 1–109, 2014):

Group 1: the agent is carcinogenic to humans;

Group 2A: the agent is probably carcinogenic to humans;

Group 2B: the agent is possibly carcinogenic to humans;

Group 3: the agent is not classifiable as to its carcinogenicity to humans.

**Table 3:** Oxidative damage of DNA/RNA, BPDE-DNA adducts, and chromosomal aberrations in children treated by GT.

Oxidative damage of DNA and RNA^a^		Significance of differences
Before GT	After GT	
(*n* = 19)	(*n* = 19)	
2557	2687	*P* < 0.05
(2008–2789)	(2146–3238)	

BPDE-DNA adducts^b^		Significance of differences
Before GT	After GT	
(*n* = 14)	(*n* = 14)	

5.65	7.2	*P* < 0.01
(4.97–7.3)	(5.35–7.9)	

Chromosomal aberration in peripheral lymphocytes^c^		Significance of differences
Before GT	After GT	
(*n* = 19)	(*n* = 19)	

Structurally aberrated cells		
0.0	1.0	*P* < 0.01
(0.0-0.0)	(0.0–1.0)	

Numerically aberrated cells		
0.0	0.0	NS
(0.0-0.0)	(0.0–1.0)	

Total number of aberrated cells		
0.0	1.0	*P* < 0.001
(0.0-0.0)	(0.0–1.0)	

^a^Oxidative damage of DNA and RNA is presented as the sum of three oxidized guanine species in serum: 8-hydroxy-2′-deoxyguanosine from DNA, 8-hydroxyguanosine from RNA, and 8-hydroxyguanine from either DNA or RNA. The levels are expressed as pg of the sum of oxidized guanine species per mL of serum. The average concentration is presented as median and lower-upper quartile because of nonnormal data distribution; *P*: Wilcoxon matched-pairs test.

^b^The levels are expressed as ng of BPDE-DNA adducts per *µ*g of DNA and the average concentration is presented as median and lower-upper quartile because of nonnormal data distribution; *P*: Wilcoxon matched-pairs test.

^c^The levels are expressed as percentage of structurally aberrated cells (SAC), numerically aberrated cells (NAC), and total number of aberrated cells (ABC). The average levels of aberration are presented as median and lower-upper quartile because of nonnormal data distribution; *P*: Wilcoxon matched-pairs test; NS: nonsignificant difference.

**Table 4:** The associations between monitored biomarkers (levels after GT) and important characteristics of exposure (Spearman Rank Order Correlations).

	Total duration of GT (days)	Time of UVR exposure (days)	Daily topical application of CCT ointment (% of body surface)
Oxidative damage of DNA and RNA^a^	NS	NS	NS
BPDE-DNA adducts^b^	NS	NS	*r* = 0.62 *P* < 0.05
Total number of aberrated cells^c^	NS	*r* = 0.55 *P* < 0.05	NS
PASI score^d^	NS	NS	*r* = 0.65 *P* < 0.01

^a^Unit: pg of the sum of three oxidized guanine species per ml of serum.

^b^Unit: ng of BPDE-DNA adducts per *µ*g of DNA.

^c^Unit: percentage of total number of aberrated cells (ABC).

^d^Dimensionless.
